# Frequency profiles of muscle-strengthening exercise identified using three national surveillance measures in a convenience sample of adults

**DOI:** 10.3389/fspor.2026.1864851

**Published:** 2026-09-30

**Authors:** Christina Johnson, Joshua Haworth, Elise Brown

**Affiliations:** 1 Department of Human Movement Science, Oakland University, Rochester, MI, United States; 2 Department of Public and Environmental Wellness, Oakland University, Rochester, MI, United States

**Keywords:** guideline adherence, latent profile analysis, physical activity surveillance, public health, resistance training, self-Report measurement

## Abstract

Muscle-strengthening exercise (MSE) is crucial for health, with current guidelines recommending engaging in MSE at least two times per week. In the US, the National Health and Nutrition Examination Survey (NHANES), National Health Interview Survey (NHIS), and Behavioral Risk Factor Surveillance System (BRFSS) assess MSE frequency, however, research on frequency-based adherence is limited. The purpose of this study is to identify and describe subgroups of MSE participation based on frequency responses to NHANES, NHIS, and BRFSS MSE survey items. In this cross-sectional analysis, a convenience sample of participants (*n* = 118 [male = 54, female = 64], mean age [±standard deviation] = 27.0 ± 10.7 years) completed an MSE questionnaire at two time points approximately one week apart that included MSE frequency items from the NHANES, NHIS, and BRFSS. Latent profile analysis (LPA) was conducted on data from the first time point to identify latent MSE frequency subgroups, while bootstrap likelihood ratio tests (BLRT; *B* = 5,000) were utilized to help determine the optimal number of profiles. The LPA indicated a three-profile solution as the best overall fit based on the difference in Bayesian information criterion, strong relative entropy (0.980), and adequate profile distribution (*p*1 = 28.8%, *p*2 = 50.0%, *p*3 = 21.2%). The BLRT supported the three-profile solution, with the addition of a third profile significantly improving fit compared to the two-profile model (*p* < 0.001). Profiles were labeled based on the average times per week of MSE. They included, (1) Minimal Participation With No Guideline Adherence (0.23 ± 0.43 times per week), (2) Moderate Participation With Guideline Adherence (3.30 ± 0.74 times per week), and (3) High Participation With Guideline Adherence (5.53 ± 0.66 times per week). Three distinct MSE frequency profiles were identified in this convenience sample, with 71.2% of participants classified into guideline-adherent profiles, substantially higher than national estimates of <30%. Within this sample, the identification of two guideline-adherent frequency profiles suggests that binary classification may mask variability in MSE frequency among individuals meeting current guidelines. These findings should be validated in nationally representative samples.

## Introduction

Muscle-strengthening exercise (MSE) is essential for promoting general health and preventing numerous chronic diseases including cardiovascular disease (CVD) (1) and type 2 diabetes (2). Benefits of regular MSE include improved cardiometabolic health, musculoskeletal health, functional capacity, and reduced mortality risk (3). Despite the plethora of health benefits that MSE can provide, MSE guideline adherence is low within the general population (<30%) (4, 5). Part of this may be due to traditional MSE organizational guidelines being relatively vague or nonunanimous, both for the general population and those with chronic diseases (1, 5–7).

The World Health Organization (WHO) states that adults (aged 18–64 years) should engage in MSE at least 2 days per week, involving all major muscle groups (1, 5). The American College of Sports Medicine (ACSM) and The United States Department of Health & Human Services additionally recommend that MSE should be performed at moderate or greater effort (6, 7).

A recent ACSM positional stand further shifts away from direct training variables toward individualized programming (8). This emphasizes effort-based approaches (i.e., repetitions in reserve), flexible modality selection (i.e., bodyweight, bands, free weights), broader conceptual definitions of resistance training movements (i.e., push and pull classifications) (9), and minimal effective dosing strategies (8). While these updates may improve accessibility and participation, they also introduce additional variability that may challenge the consistent assessment and comparison of MSE behaviors across large-scale surveillance systems (10).

The current state of MSE guidelines highlights the importance of accurately surveying and quantifying MSE behavior in both the general population and clinical populations, including individuals with CVD and T2D (5). Multiple self-report surveys have been developed and implemented at large scales to help determine frequency of MSE at the population level (11–14). Within the United States, these include the National Health and Nutrition Examination Survey (NHANES) (11), National Health Interview Survey (NHIS) (13), and Behavioral Risk Factor Surveillance System (BRFSS) (12). However, it is important to note that these surveys differ in ways that may meaningfully influence the interpretation of MSE behavior.

These three surveys vary in how they request recall and define/exemplify MSE, particularly in the terminology and examples provided to respondents. The NHANES uses terms such as “*lifting weights, push-ups, or sit-ups*” (11). The NHIS uses terminology such as “*lifting weights or doing calisthenics*” (13). The BRFSS uses verbiage such as “*using your body weight like yoga, sit-ups or pushups and those using weight machines, free weights, or elastic bands*” (12). Moreover, the BRFSS allows participants to report frequency in either times per week or times per month, while the NHANES and NHIS require reporting in times per week (11–13). These collective differences may lead to variability in how respondents interpret or report MSE participation.

Current MSE frequency research often classifies individuals as either “guideline adherent” or “not guideline adherent” based on whether they meet the minimum recommended weekly frequency (4). While binary classification is useful for estimating prevalence and monitoring public health trends, classifying MSE frequency as guideline adherent or non-adherent reduces the information available to characterize variation among individuals within the same adherence category (15). Separately, health behaviors are multifaceted and may reflect broader patterns across multiple lifestyle behaviors (16, 17). Therefore, MSE participation may vary based on multiple factors such as demographics (i.e., age, sex, race, and education) and lifestyle behaviors, which may not be captured through binary guideline adherence classifications. As a result, a more detailed examination of behavioral classification may provide meaningful insight beyond adherence prevalence alone.

Although research on MSE behavior has examined populations ranging from adolescents (18) to the general adult population (4), there is limited research examining frequency behavioral patterns. Current literature focuses on yes/no adherence, however, yes/no response formats can inflate agreement rates and obscure variability in behavior (15). Thus, the use of frequency-based measures may provide a more detailed characterization of MSE adherence patterns (15). Additionally, more in-depth analysis of MSE frequency may allow for the identification of latent freuqnecy subgroups within the traditional binary classification, which often assumes populations within the same classifications are the same.

Hence, it is unknown if MSE frequency profiles exist within those who engage in MSE. Therefore, the purpose of the current study is to identify and describe subgroups of MSE participation based on frequency responses to NHANES, NHIS, and BRFSS MSE survey items. Given the exploratory nature of this secondary analysis, no *a priori* hypothesis was specified regarding the number or characteristics of the MSE frequency profiles that would emerge.

## Methods

This data sample was curated from a separate recruitment effort for a reliability (*n* = 162) portion of a larger study (19). Methods and results from this parent study have not been published other than said conference abstract, though the current analysis does not depend on the broader aims of the study. All procedures performed in this study were in accordance with the ethical standards of the institutional review board and with the 1964 Helsinki declaration and its later amendments or comparable ethical standards. The data were analyzed in this cross-sectional study examining MSE frequency responses across three national surveillance systems. The parent reliability study utilized convenience sampling with self-selection, with participants recruited by health science undergraduate and graduate students. Informed consent was obtained from all individual participants included in the study. Inclusion criteria required participants to be aged 18–65 years, not pregnant or planning to become pregnant, and be able to complete an online survey in the English language.

Figure 1 showcases participant inclusion, exclusion, and progression through the study. Participants completed the Muscle-Strengthening Exercise Survey (MSES) at two separate time points approximately a week apart. However, only the first time point was used for analysis in the current study. The MSES was divided into three sections: (1) Muscle-Strengthening Exercise Questionnaire-Modified (19), (2) National Survey Muscle-Strengthening Exercise Items from National Surveys, and (3) About You section. Section two included MSE frequency survey items from the NHANES, NHIS, and BRFSS, while section three included demographic questions (i.e., age, race, sex, education), and these three MSE frequency surveys were utilized in tandem to identify distinct profiles of guideline adherence. Data from the National Survey Items and the About You section were used in the current study for analysis. Questions were worded in a manner where participants selected “yes” or “no” to participating in MSE and then manually entered the number of times per week or times per month. Exact wording of the MSE frequency items and the response options are available via Supplementary Appendix A.

**Figure 1:**
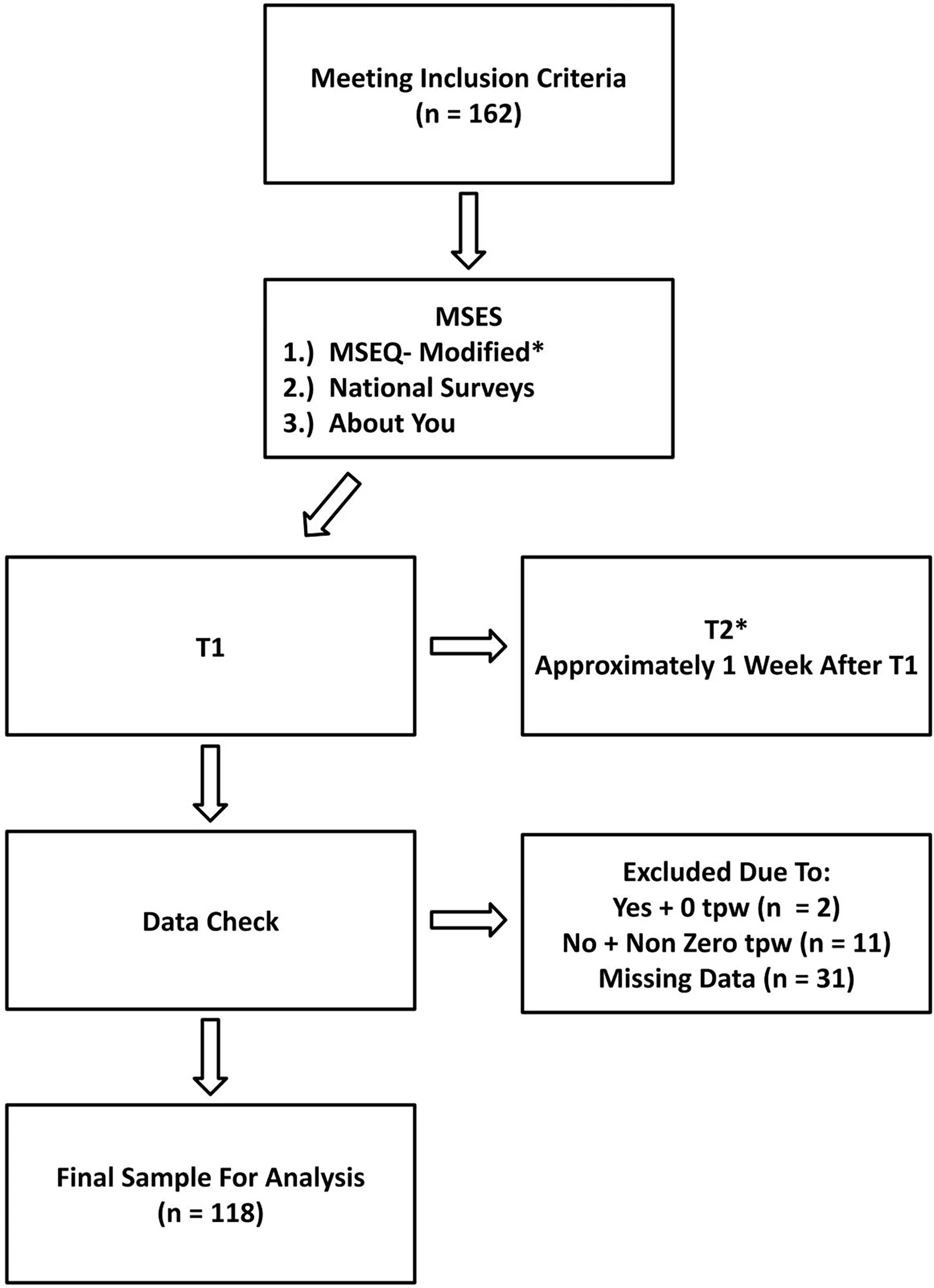
Participant flow diagram for latent profile analysis. MSEQ, modified, muscle-strengthening exercise questionnaire modified; MSES, muscle-strengthening exercise survey; TPW, times per week; TP1, timepoint 1; TP2, timepoint 2. *Not analyszed within this study.

Data were checked for inconsistent responses or missing MSE frequency data across the three surveys resulting in exclusion from analysis (*n* = 44). Participants with missing data on one or more of the three MSE frequency indicators were excluded from the latent profile analysis using complete-case analysis (*n* = 31). Imputation was not performed because the three MSE frequency measures constituted the profile-defining indicators, therefore, imputing missing values would require estimating the response patterns used directly to derive the latent profiles. Conditions for exclusions were as follows: (1) if a participant reported “no” (i.e., they did not engage in MSE) but manually entered a non-zero number of times per week (*n* = 11), (2) if a participant reported “yes” (i.e., they did engage in MSE) but manually entered zero times per week (*n* = 2), or (3) if any survey data were missing (*n* = 31). Demographic statistics for excluded participants are reported within Table 1. After exclusions, 118 participants (27.0 ± 10.7 years, female = 64, male = 54) remained for analysis.

**Table 1:** Whole sample demographics vs. ACS 1-year estimates (2024) demographic and housing estimates (DP05) and ACS 1-year estimates (2024) education attainment (S1501).

Demographic	Excluded sample (*n* = 44)	Current sample (*n* = 118)	United States Census
Age (years)			
Mean ± SD	25.14 ± 8.92	27.0 ± 10.7	–
Median (IQR)	22 (IQR: 20–25)	23 (IQR: 20–27)	39.2
Sex			
Female	68.2% (*n* = 30)	54.2% (*n* = 64)	50.5%
Male	31.8% (*n* = 14)	45.8% (*n* = 54)	49.5%
Race/Ethnicity			
White	63.6% (*n* = 28)	66.1% (*n* = 78)	58.9%
Black/African American	–	5.1% (*n* = 6)	12.4%
Asian	4.5% (*n* = 2)	11.9% (*n* = 14)	6.3%
Middle Eastern/North African	18.2% (*n* = 8)	10.2% (*n* = 12)	Not separately reported
Other	–	2.5% (*n* = 3)	1.5%
Prefer not to say	2.3% (*n* = 1)	0.8% (*n* = 1)	–
Missing	9.1% (*n* = 4)	3.4% (*n* = 4)	–
Hispanic/Latino/Spanish	2.3% (*n* = 1)	–	–
Ethnicity: Hispanic	–	–	18.7%
Education			
1–11th Grade	–	0.8% (*n* = 1)	–
HS Diploma	9.1% (*n* = 4)	12.7% (*n* = 15)	–
GED	–	0.8% (*n* = 1)	–
Less than 1 year college	–	4.2% (*n* = 5)	–
1+ Year of college, no degree	36.4% (*n* = 16)	28.0% (*n* = 33)	–
Associate's	9.1% (*n* = 4)	8.5% (*n* = 10)	–
Bachelor's	34.1% (*n* = 15)	28.8% (*n* = 34)	–
Master's	6.8% (*n* = 3)	11.9% (*n* = 14)	–
Professional	–	3.4% (*n* = 4)	–
Doctorate	4.5% (*n* = 2)	0.8% (*n* = 1)	–
High school or higher	100.0% (*n* = 44)	99.2% (*n* = 117)	89.9%
Bachelor's or higher	45.5% (*n* = 20)	44.9% (*n* = 53)	36.8%

Hispanic/Latino/Spanish was included as a response option within the demographic race/ethnicity item administered to participants and is presented as originally collected. GED, general educational development; HS, high school; IQR, interquartile range; SD, standard deviation. Data is presented as % (*n*) unless otherwise indicated.

A sensitivity analysis was conducted to assess the influence of excluding participants with inconsistent data responses. The thirteen participants with said inconsistent responses were retained (*n* = 131), with the numeric frequency response used for analysis, and the correlations and LPA procedures were repeated using the same approach as the primary analysis.

Pearson correlation coefficients were calculated among the NHANES, NHIS, and BRFSS MSE frequency measures to quantify the relationships among the indicators. Latent profile analysis (LPA) was conducted in R version 4.5.1 using the tidyLPA package (version 2.0.2) with model estimation performed through mclust (version 6.1.3). LPA models specifying one to five latent profiles were estimated to identify the optimal profile solution. Models were estimated assuming equal variances across profiles and zero covariances among indicators (20). Methodology for determining the best model fit was: (1) smaller improvements in Bayesian information criterion (ΔBIC) (21, 22), (2) acceptable relative entropy values (20), (3) reasonable profile size distribution, (4) smaller improvements in log likelihood values (ΔLL) (23), and (5) parsimony (24, 25). After the initial LPA was run, the bootstrap likelihood ratio test (BLRT) was used to compare model fit with 5,000 bootstrap samples and accepted if *p* < 0.05 (26).

To evaluate the robustness of the profile solution to the assumption of local independence, a sensitivity analysis was conducted in which covariances among the three MSE frequency indicators were estimated but constrained to be equal across profiles. One- through five-profile solutions were re-estimated under this alternative specification using the same model-selection criteria as the primary analysis. A more flexible specification allowing both variances and covariances to vary across profiles was also examined, however, models containing two or more profiles could not be estimated.

Next, the final model was chosen considering the stated criteria while prioritizing diminishing improvements and parsimony. Comparison of the current study sample to the 2024 U.S. population (27, 28) was performed to assess population representativeness. Demographic characteristics were also compared between participants included in the analytic sample (*n* = 118) and those excluded from the analysis (*n* = 44). Age was compared using Welch's independent-samples *t*-test, sex using the chi-square test of independence, and race/ethnicity and education using Fisher's exact tests due to small expected cell counts. Participants were split into their assigned profiles where demographic analysis was conducted between the profiles. As age is a continuous variable, a one-way analysis of variance (ANOVA) was used to test for differences between profiles. Homogeneity of variance was assessed using Levene's test, with statistical significance set at *p* < 0.05. When the assumption of homogeneity of variance was violated (*p* < 0.05), Welch's one-way ANOVA was used for the comparison. Effect size for age was assessed using omega-squared. The chi-square test of independence was used for the categorical variables of sex and education. Education was collapsed into two categories of “*Associate's or Below*” and “*Bachelor's or Higher*” to ensure aligned comparison between the U.S. census data and to ensure greater than five counts in each cell. Fisher's exact test was utilized for the categorical variable of race as there were multiple cells with less than five counts. Effect sizes for categorical comparisons were assessed using Cramér's *V*.

This study was reported in accordance with the Strengthening the Reporting of Observational Studies in Epidemiology (STROBE) guidelines for cross-sectional studies, with the completed checklist provided in Supplementary Appendix B.

## Results

Participant demographics are described in Table 1. Compared with U.S. population estimates, the current convenience sample had a similar sex distribution but was younger, more highly educated, and differed in racial composition (27, 28). Additionally, Middle Eastern/North African participants were reported as a distinct racial category in the present study, whereas this group has historically been classified within the White racial category in the U.S. Census data (27, 29). These comparisons should be interpreted cautiously because the available Census age, race, and education estimates do not directly align with the study inclusion criteria of adults aged 18–65 years.

The three MSE frequency measures demonstrated strong intercorrelations (*r* = 0.879–0.945, all *p* < 0.001; Table 2), indicating strong associations among the indicators used in the latent profile analysis. Profile-specific means were also highly similar across the three survey measures, suggesting that the latent profiles primarily reflected differences in overall weekly MSE frequency. Accordingly, the identified profiles should be interpreted as minimal-, moderate-, and high-frequency participation groups rather than qualitatively distinct behavioral patterns. To determine the latent profiles, 1–5 models were tested.

**Table 2:** Pearson correlations Among muscle-strengthening exercise frequency measures.

Survey	NHANES	NHIS	BRFSS
NHANES	–	0.945 [0.922, 0.962]	0.879 [0.830, 0.915]
NHIS		–	0.898 [0.857, 0.928]
BRFSS			–

*N* = 118. Values are Pearson correlation coefficients (*r*) with 95% confidence intervals in brackets. All correlations were significant at *p* < .001. BRFSS, behavioral risk factor surveillance system; NHANES, national health and nutrition examination survey; NHIS, national health interview survey.

As shown in Table 3, formal fit criteria continued to favor solutions with additional profiles, as the BIC decreased across each successive model and the BLRT remained significant (*p* < 0.001). The number of freely estimated parameters increased from 6 in the 1-profile solution to 10, 14, 18, and 22 in the 2- through 5-profile solutions, respectively. However, the magnitude of improvement in model fit diminished with additional profiles. The 3-profile solution was retained based on the elbow in model fit, profile size, interpretability, and parsimony. Specifically, this was supported by (1) diminishing improvements in BIC across successive models (largest improvement observed between the 1- and 2-profile solutions [ΔBIC = 247.680]), followed by smaller improvements for the 2- to 3-profile (ΔBIC = 147.583), 3- to 4-profile (ΔBIC = 61.188), and 4- to 5-profile solutions (ΔBIC = 3.772) (21, 22), (2) acceptable relative entropy (0.980) (20), (3) reasonable profile size distribution (profile 1 size = 28.8%, profile 2 size = 50.0%, profile 3 size = 21.2%), (4) diminishing improvements in log-likelihood across successive models (largest increase observed between the 1- and 2-profile solutions [ΔLL = 133.381]), followed by smaller improvements for the 2- to 3-profile (ΔLL = 83.333), 3- to 4-profile (ΔLL = 40.135), and 4- to 5-profile solutions (ΔLL = 11.427) indicating diminishing returns with additional profiles (23). Moreover, this pattern of diminishing improvement was also present with the AIC and SABIC (30).

**Table 3:** Fit statistics for latent profile analysis models.

Specification	Profiles	Parameters	LL	ΔLL	AIC	BIC	ΔBIC	SABIC	Entropy	Smallest profile %	BLRT (*p*)
Zero covariance (primary)	1	6	−759.868	–	1,531.737	1,548.361	–	1,529.394	–	100%	–
	2	10	−626.487	133.381	1,272.974	1,300.681	247.680	1,269.068	0.928	33.9%	*p* < 0.001
	3	14	−543.154	83.333	1,114.308	1,153.098	147.583	1,108.840	0.980	21.2%	*p* < 0.001
	4	18	−503.019	40.135	1,042.038	1,091.910	61.188	1,035.007	0.962	12.7%	*p* < 0.001
	5	22	−491.592	11.427	1,027.183	1,088.138	3.772	1,018.591	0.947	11.0%	*p* < 0.001
Equal covariance (sensitivity)	1	12	−528.214	–	1,080.427	1,113.676	–	1,075.741	–	100%	–
	2	16	−516.043	12.171	1,064.086	1,108.417	5.259	1,057.837	0.858	33.1%	*p* < 0.001
	3	20	−496.730	19.313	1,033.461	1,088.874	19.543	1,025.649	0.937	22.0%	*p* < 0.001
	4	24	−483.396	13.334	1,014.793	1,081.289	7.585	1,005.419	0.938	16.1%	*p* < 0.001
	5	28	−457.939	25.458	971.877	1,049.457	31.832	960.942	0.956	0.8%	*p* < 0.001

AIC, Akaike information criterion; BIC, Bayesian information criterion; BLRT, bootstrap likelihood ratio test; LL, log-likelihood; SABIC, sample-size adjusted Bayesian information criterion.

When covariances among the three MSE frequency indicators were estimated but constrained to be equal across profiles, the overall frequency-ordered profile structure was preserved, although the optimal number of profiles was sensitive to model specification (Table 3). The three-profile solution demonstrated high entropy (0.937) and adequate profile sizes (smallest profile = 22.0%), however, the four-profile solution demonstrated lower AIC, BIC, and SABIC values, comparable entropy (0.938), and adequate profile sizes (smallest profile = 16.1%). The four-profile solution consisted of minimal-, moderate-, moderately high-, and high-frequency profiles, with model-estimated frequencies ranging from 0.12–0.24, 2.66–3.12, 3.93–4.11, and 5.28–5.84 times per week across the three indicators, respectively. Cross-classification indicated that the additional profile primarily reflected further differentiation of participants classified in the moderate-frequency profile in the primary three-profile solution. Although the five-profile model demonstrated lower information criteria, its smallest profile contained only one participant (0.8%) and therefore did not meet the prespecified minimum profile-size criterion.

Formal comparisons between the analyzed sample (*n* = 118) and the excluded sample (*n* = 44) indicated no significant differences in age [Welch's *t*(89.68) = 1.12, *p* = 0.267], sex [*χ*^2^(1) = 2.02, *p* = 0.155], race/ethnicity (Fisher's exact *p* = 0.112), or education (Fisher's exact *p* = 0.557). Sensitivity analyses retaining the thirteen participants with inconsistent responses produced lower, but still strong, correlations among the three MSE frequency measures (*r* = 0.776–0.890). The three-profile structure remained similar, with minimal (32.8%; *n* = 43), moderate (48.1%; *n* = 63), and high (19.1%; *n* = 25) frequency profiles averaging 0.40 ± 0.55, 3.27 ± 0.73, and 5.53 ± 0.66 times per week, respectively. The proportion classified into guideline-adherent profiles decreased from 71.2% in the primary analysis to 67.2% in the sensitivity analysis.

The retrained three-profile solution required further analysis from the LPA to understand each profile separately. Data such as individual participant profile distribution as well as averaged MSE times per week were extracted. According to Table 4, Profile 1 consisted of 28.8% (*n* = 34) of the total sample and averaged 0.23 ± 0.43 times per week of MSE participation. Profile 2 consisted of 50.0% (*n* = 59) of the total sample and averaged 3.30 ± 0.74 times per week of MSE participation. Profile 3 consisted of 21.2% (*n* = 25) of the total sample and averaged 5.53 ± 0.66 times per week of MSE participation. Based on averaged MSE participation, these profiles were labeled as (1) *Minimal Participation With No Guideline Adherence (MPNGA)*, (2) *Moderate Participation With Guideline Adherence (MPGA)*, and (3) *High Participation With Guideline Adherence (HPGA)*. Labeling was derived from relative participation and if at least two times per week of MSE participation was achieved. The three profiles demonstrate MSE frequency patterns across the NHANES, NHIS, and BRFSS measures, with MPNGA consistently below and MPGA and HPGA above the guideline threshold of at least two times per week (Figure 2). Demographics of each profile are shown in Table 5.

**Table 4:** Muscle-strengthening exercise frequency by survey measure and latent profiles.

Survey	MPNGA (*n* = 34)	MPGA (*n* = 59)	HPGA (*n* = 25)
	Mean ± SD [95% CI]	Mean ± SD [95% CI]	Mean ± SD [95% CI]
NHANES	0.24 ± 0.55 [0.04, 0.43]	3.19 ± 1.03 [2.92, 3.45]	5.56 ± 0.71 [5.27, 5.85]
NHIS	0.18 ± 0.46 [0.02, 0.34]	3.25 ± 0.78 [3.05, 3.46]	5.72 ± 0.54 [5.50, 5.94]
BRFSS	0.26 ± 0.51 [0.09, 0.44]	3.46 ± 1.07 [3.18, 3.74]	5.32 ± 1.07 [4.88, 5.76]
Grand mean	0.23 ± 0.43 [0.07, 0.38]	3.30 ± 0.74 [3.11, 3.49]	5.53 ± 0.66 [5.26, 5.81]

BRFSS, behavioral risk factor surveillance system; CI, confidence interval; NHANES, national health and nutrition examination survey; NHIS, national health interview survey; HPGA, high participation with guideline adherence; MPGA, moderate participation with guideline adherence; MPNGA, minimal participation with no guideline adherence; SD, standard deviation.

**Figure 2:**
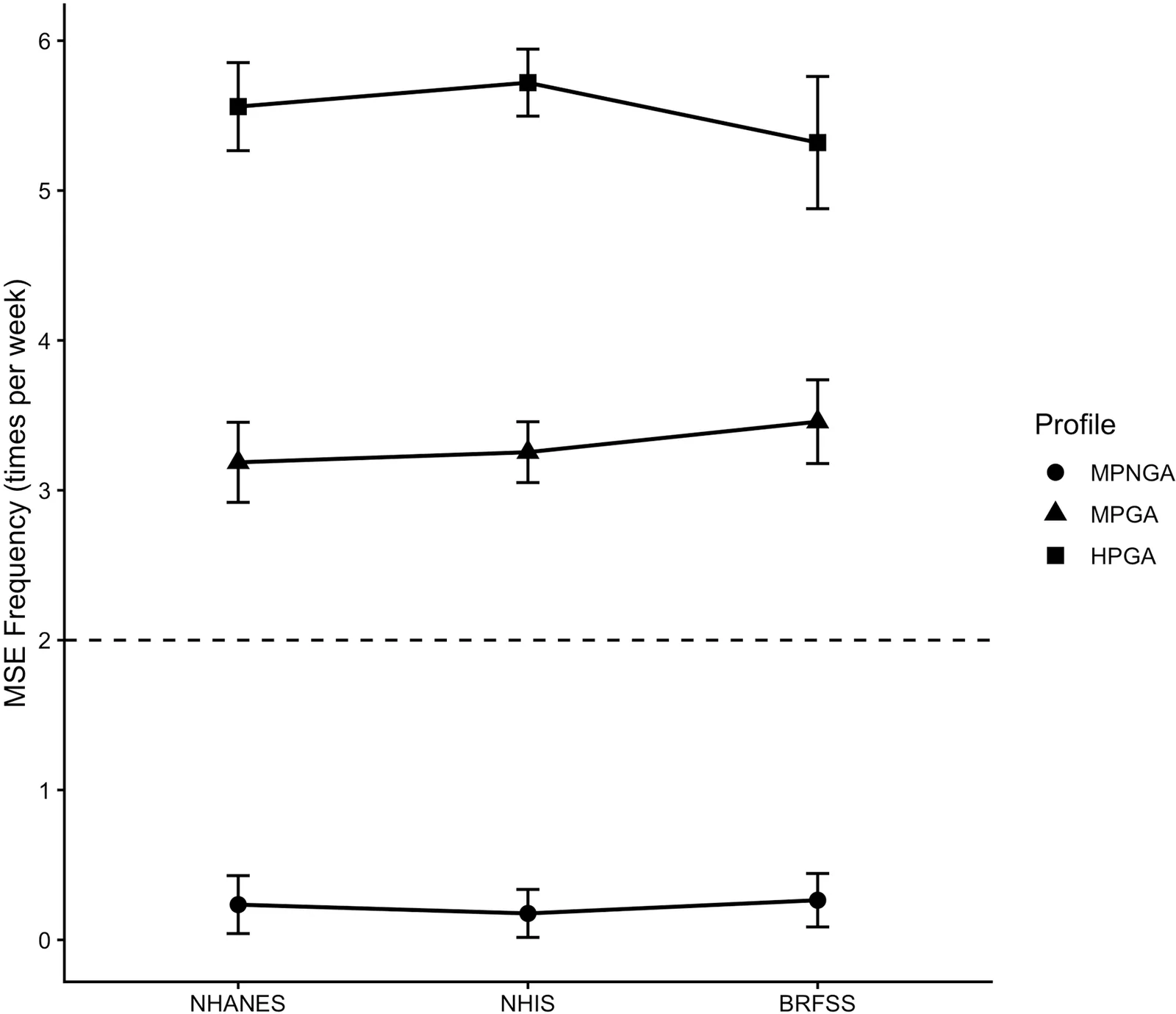
Muscle-strengthening exercise frequency across survey measures by latent profiles. Points represent mean MSE frequency and error bars represent 95% confidence intervals. The horizontal dashed line represents the MSE guideline threshold of ≥2 times per week. BRFSS, behavioral risk factor surveillance system; NHANES, national health and nutrition examination survey; NHIS, national health interview survey; HPGA, high participation with guideline adherence; MPGA, moderate participation with guideline adherence; MPNGA, minimal participation with no guideline adherence; MSE, muscle-strengthening exercise.

**Table 5:** Demographics of three identified profiles.

Demographic	MPNGA (*n* = 34)	MPGA (*n* = 59)	HPGA (*n* = 25)
Age (years)	30.4 ± 12.9	25.4 ± 8.9	26.2 ± 10.5
*p* = 0.139			
*ω*^2^ = 0.026			
Sex			
*p* = 0.136			
*V* = 0.184			
Female	50.0% (*n* = 17)	62.7% (*n* = 37)	40.0% (*n* = 10)
Male	50.0% (*n* = 17)	37.3% (*n* = 22)	60.0% (*n* = 15)
Race/ Ethnicity			
*p* = 0.681			
*V* = 0.199			
White	61.8% (*n* = 21)	64.4% (*n* = 38)	76.0% (*n* = 19)
Black/African American	2.9% (*n* = 1)	6.8% (*n* = 4)	4.0% (*n* = 1)
Asian	20.6% (*n* = 7)	10.2% (*n* = 6)	4.0% (*n* = 1)
Middle Eastern/North African	11.8% (*n* = 4)	10.2% (*n* = 6)	8.0% (*n* = 2)
Other	–	5.1% (*n* = 3)	–
Prefer not to say	–	1.7% (*n* = 1)	–
Missing data	2.9% (*n* = 1)	1.7% (*n* = 1)	8.0% (*n* = 2)
Education^a^			
*p* = 0.427			
*V* = 0.120			
1–11th Grade	–	1.7% (*n* = 1)	–
HS Diploma	23.5% (*n* = 8)	10.2% (*n* = 6)	4.0% (*n* = 1)
GED	–	–	4.0% (*n* = 1)
Less than one year college	2.9% (*n* = 1)	6.8% (*n* = 4)	–
One or more year college no degree	20.6% (*n* = 7)	32.2% (*n* = 19)	28.0% (*n* = 7)
Associate's	2.9% (*n* = 1)	10.2% (*n* = 6)	12.0% (*n* = 3)
Bachelor's	29.4% (*n* = 10)	28.8% (*n* = 17)	28.0% (*n* = 7)
Master's	20.6% (*n* = 7)	5.1% (*n* = 3)	16.0% (*n* = 4)
Professional	–	5.1% (*n* = 3)	4.0% (*n* = 1)
Doctorate	–	–	4.0% (*n* = 1)
Associate's or Below^a^	50.0% (*n* = 17)	61.0% (*n* = 36)	48.0% (*n* = 12)
Bachelor's or Higher^a^	50.0% (*n* = 17)	39.0% (*n* = 23)	52.0% (*n* = 13)

Hispanic/Latino/Spanish was included as a response option within the demographic race/ethnicity item administered to participants and is presented as originally collected. GED, general educational development; HPGA, high participation with guideline adherence; HS, high school; MPGA, moderate participation with guideline adherence; MPNGA, minimal participation with no guideline adherence.

a The two categories of “Associate's or Below” and “Bachelor's or Higher” were used for statistical analysis instead of all separate education categories for the usage of Chi-square to ensure no cells had less than 5 counts.

Levene's test indicated that the assumption of homogeneity of variance was violated (*p* = 0.018). Therefore, age differences across the three latent profiles were examined using Welch's one-way ANOVA. No significant differences in age were observed across profiles, Welch's *F*_(2, 53.23)_ = 2.05, *p* = 0.139, *ω*^2^ = 0.026. According to Table 5, no significant differences in age, sex, race, or education level were observed across the three profiles (all *p* ≥ 0.136).

## Discussion

The current study sought to identify MSE frequency profiles of MSE adherence. Three profiles were identified based on MSE frequency responses to the NHANES, NHIS, and BRFSS within the MSES. Previous literature indicates that health behaviors are multidimensional (17), however to our knowledge, this is the first study to use LPA to identify frequency subgroups of MSE behavior.

The three surveillance measures were highly correlated, yet they were not identical. This is evident as correlations are below 1.00 and small differences in profile-specific means across surveys are present. Including all three measures therefore allowed the latent profile analysis to incorporate variation across commonly used national surveillance instruments rather than relying on a single survey measure.

These findings demonstrate that there is statistical separation between the three profiles (*MPNGA*, *MPGA*, and *HPGA*). Because the latent profiles were derived from weekly MSE frequency, the resulting groups should be interpreted as frequency-based participation categories rather than distinct behavioral phenotypes. Although this frequency-based separation is inherent to the modeling approach, it highlights that MSE participation is more appropriately viewed as a continuum than as a binary characteristic.

Within the general population, MSE guideline adherence is known to be low (<30%) (4, 5). However, based on the results in the current sample, 71.2% of the sample is guideline adherent. This difference may be due to several factors. First, the present study used data that was derived from a convenience sample recruited by undergraduate and graduate health science students, rather than a nationally representative sample (4, 5), which may explain the higher adherence. Alternatively, differences in how participants interpret and respond to national MSE surveillance questions may contribute to differences in estimated guideline adherence (31, 32). Because this study did not include an objective measure of MSE participation, it cannot distinguish between these explanations. These findings emphasize the need for standardization or alignment of national survey MSE items which may improve accuracy and cross-survey comparability. Furthermore, demographic characteristics may need to be considered when interpreting surveillance data (33).

Ren et al. and Ji et al. demonstrated that demographic characteristics of being male, younger, and highly educated are associated with increased likelihood of being MSE guideline adherent in the general population (4, 33). Statistically, this is not consistent with results in the current study as there were no statistically significant demographic differences between the three profiles. However, in the *HPGA* class, 60.0% (*n* = 15) men comprised the profile while 62.7% (*n* = 37) women made up the *MPGA* class. A similar trend was observed for age as the oldest mean age between the three profiles was the *MPNGA* group (30.4 ± 12.9 years old). Moreover, 14.4% (*n* = 17) of the total sample was classified in the *non-guideline adherence* group and had an education level above an associate's degree, whereas 30.5% (*n* = 36) of the total sample was classified into either *guideline adherent* groups and had an education level above an associate's degree.

Current MSE guideline research does not directly investigate MSE frequency, but rather uses the variable to determine guideline adherence (at least two times of MSE per week) (4, 5, 18). Both the moderate- and high-frequency profiles exceeded the current MSE frequency guideline of at least two sessions per week. However, LPA categorized their frequency into two distinct groups, indicating their frequency behaviors are statistically different from one another. This conclusion poses the question, *If there are statistically different frequency groups within a “guideline adherent” category, does the current binary classification system oversimplify adherence and mask important variability in exercise behavior?* A similar approach is used in muscular function assessment, where age- and sex- specific handgrip strength percentiles provide graded reference values that preserve information that would be lost through single threshold classification (34). Therefore, the current study shows that MSE frequency behavior is more than a simple binary classification, which further highlights the need for more research in MSE participation and demographic relationships (35).

An important consideration when interpreting the identified profiles is the substantially higher prevalence of MSE guideline adherence in the current sample compared with national estimates. In the present study, 71.2% of participants were classified into the moderate- or high-frequency profiles and therefore reported MSE frequencies consistent with current guidelines, whereas national surveillance data indicate that fewer than 30% of U.S. adults meet MSE recommendations (4, 5). This discrepancy may reflect the convenience sampling strategy, as participants were recruited through undergraduate and graduate health science students and may have been more physically active or interested in exercise than the general population. Consequently, the relative size and potentially the structure of the identified frequency profiles may differ in a nationally representative sample. In particular, a representative sample with a substantially greater proportion of adults not meeting MSE guidelines may yield different profile proportions or additional differentiation within lower-frequency MSE participation. Replication in a nationally representative sample is therefore necessary to determine whether the frequency profiles identified in the present study generalize to the broader U.S. adult population.

Future research may include LPA and other data-driven analytic approaches to nationally representative datasets to identify more in-depth frequency characteristics that can be applied to the general population. Recent work has highlighted the potential of machine learning methods to identify meaningful patterns within large physical activity datasets while also emphasizing the importance of validating data-driven subgroup classifications and evaluating their practical utility for public health surveillance (36). Moreover, clinical applications should be explored within both guideline adherent groups as the HPGA group might be at an increased risk for overadherence or overtraining potentially resulting in higher injury rates (37). Prescriptive MSE recommendations suggest two to three sessions per week across all major muscle groups, providing additional context for the HPGA profile's average frequency of 5.53 times per week (8). However, because training volume, intensity, recovery, and muscle groups trained were not assessed, higher MSE frequency should not independently be interpreted as overtraining. Moreover, it may be beneficial to investigate other biopsychosocial factors that influence MSE behaviors such as physical health status, health literacy, socioeconomic status, geographical locations (e.g., urban vs. rural), and marital or relationship status (38, 39). Lastly, to help minimize self-reporting biases, a comparison can be conducted between self-report frequency and objective measures of MSE participation, such as video-based behavioral observation (40).

Multiple limitations should be considered when interpreting these findings. First, the three MSE frequency measures were highly correlated, and the resulting latent profiles primarily reflected differences in overall weekly MSE frequency rather than qualitatively distinct behavioral patterns (22, 23). Although sensitivity analyses allowing covariance among the indicators preserved the overall frequency-ordered structure, the optimal number of profiles was sensitive to model specification. Therefore, the precise number of MSE frequency profiles should be interpreted cautiously. Second, because no objective criterion measure of MSE participation was included, the present study cannot determine whether differences in reported guideline adherence reflect true differences in participation or differences in how participants interpreted and responded to the surveillance questions. Third, although data were collected at two time points as part of the reliability study, only the first assessment was included in the latent profile analysis, therefore, the present analyses should be interpreted as cross-sectional. Additionally, the latent profile analysis should be considered exploratory because it was conducted as a secondary analysis from a parent study for which the methods and results are not currently published other than in a conference abstract, and no *a priori* sample size calculation was performed. Moreover, the exclusion of participants with inconsistent data responses modestly increased the observed intercorrelations and estimated guideline adherence, as demonstrated by the sensitivity analysis. Convenience sampling through undergraduate and graduate health science students may further limit the generalizability of these findings. Finally, comparisons with U.S. Census demographics should be interpreted cautiously because the available Census age, race, and education data do not directly align with the study inclusion criteria of adults aged 18–65 years (27, 28).

This study had multiple strengths. First, three major national surveillance systems within the same sample were utilized to reduce possible measurement bias. The use of LPA was also a strength, as it allowed identification of meaningful subgroups that traditional regression would not detect. Lastly, the use of a sensitivity analysis strengthened the findings, demonstrating that the three-profile structure remained similar when participants with inconsistent data responses were retained.

## Conclusion

The current study identified three subgroups based on MSE frequency responses across a partial representation of the U.S. demographics: (1) *MPNGA*, (2) *MPGA*, and (3) *HPGA*. These findings demonstrate that MSE participation is more appropriately represented as a continuum of weekly frequency rather than a simple binary classification of guideline adherence. Accordingly, interpretation of national MSE surveillance data should recognize that individuals meeting the recommended guideline may differ substantially in participation frequency. Future research should evaluate whether incorporating frequency-based classifications into surveillance systems provides greater insight into population MSE participation patterns and improves comparisons across national surveillance measures.

## Data Availability

The raw data supporting the conclusions of this article will be made available by the authors, without undue reservation.
