# Supplementary material for: Frequency profiles of muscle-strengthening exercise identified using three national surveillance measures in a convenience sample of adults
Source: Front Sports Act Living. 2026 Sep 30;8:1864851. doi: 10.3389/fspor.2026.1864851 (PMC13662153; doi:10.3389/fspor.2026.1864851)
Supplement: Supplementary file 1 [file Supplementaryfile1.pdf]

Appendix A

Section Two: National Health Surveys

The next few questions are from national health surveys that measure different health behaviors, the Behavioral Risk Factor Surveillance System, National Health and Nutrition Examination Survey, and National Health Interview Survey.
These questions may sound similar to each other and to previous questions, but please answer them to the best of your ability.

<u>National Health and Nutrition Examination Survey</u>

Over the past 7 days, did you do any physical activities specifically designed to strengthen your muscles such as lifting weights, push-ups or sit-ups?

- ☐ Yes
- ☐ No
- ☐ Don't know/not sure

Over the past 7 days, how often did you do these physical activities? [Activities designed to strengthen your muscles such as lifting weights, push-ups or sit-ups.]

☐ Times per week

- ☐ Never
- ☐ Don't know/not sure

<u>National Health Interview Survey</u>

In the past 7 days, have you done weight lifting or other exercises to increase muscle strength?

- ☐ Yes
- ☐ No

How often do you do physical activities specifically designed to STRENGTHEN your muscles such as lifting weights or doing calisthenics?

☐ Times per week

- ☐ Never
- ☐ Don't know/not sure

Behavioral Risk Factor Surveillance System

During the past month, how many times per week OR per month did you do physical activities or exercises to strengthen your muscles?

Do not count aerobic activities like walking, running, or bicycling. Count activities using your own body weight like yoga, sit-ups or pushups and those using weight machines, free weights, or elastic bands.

ONLY SELECT ONE OF THE CHOICES BELOW.

[ ] Times per week

[ ] Times per month

- ☐ Never
- ☐ Don't know/not sure
