# Supplementary material for: Frequency profiles of muscle-strengthening exercise identified using three national surveillance measures in a convenience sample of adults
Source: Front Sports Act Living. 2026 Sep 30;8:1864851. doi: 10.3389/fspor.2026.1864851 (PMC13662153; doi:10.3389/fspor.2026.1864851)
Supplement: Supplementary file 2 [file Supplementaryfile2.pdf]

Appendix B

STROBE Statement—Checklist of Items That Should Be Included in Reports of Cross-Sectional Studies

Editable checklist for manuscript reporting

**Item**	**No.**	**Recommendation**	**Page/Section in Manuscript**
Title and abstract	1(a)	Indicate the study's design with a commonly used term in the title or the abstract	Page 2, Abstract- methods
	1(b)	Provide in the abstract an informative and balanced summary of what was done and what was found	Page 2, Abstract
Background/rationale	2	Explain the scientific background and rationale for the investigation being reported	Pages 3-5, Introduction
Objectives	3	State specific objectives, including any prespecified hypotheses	Page 5, Introduction
Study design	4	Present key elements of study design early in the paper	Page 5, Methods
Setting	5	Describe the setting, locations, and relevant dates, including periods of recruitment, exposure, follow-up, and data collection	Page 5, Methods
Participants	6(a)	Give the eligibility criteria, and the sources and methods of selection of participants	Pages 5-6, Methods
Variables	7	Clearly define all outcomes, exposures, predictors, potential confounders, and effect modifiers. Give diagnostic criteria, if applicable	Pages 6-8, Methods
Data sources/measurement	8*	For each variable of interest, give sources of data and details of methods of assessment (measurement). Describe comparability of assessment methods if there is more than one group	Pages 4 and 6, Introduction and Methods
Bias	9	Describe any efforts to address potential sources of bias	Pages 6 and 14, Methods and Discussion
Study size	10	Explain how the study size was arrived at	Pages 5-6 and 14, Methods and Discussion
Quantitative variables	11	Explain how quantitative variables were handled in the analyses. If applicable, describe which groupings were chosen and why	Pages 6-8, Methods
Statistical methods	12(a)	Describe all statistical methods, including those used to control for confounding	Pages 7-8, Methods
	12(b)	Describe any methods used to examine subgroups and interactions	Pages 7-8, Methods
	12(c)	Explain how missing data were addressed	Page 6, Methods
	12(d)	If applicable, describe analytical methods taking account of sampling strategy	N/A- convenience sample
	12(e)	Describe any sensitivity analyses	Page 6, Methods
Participants	13(a)	Report numbers of individuals at each stage of study—e.g., numbers potentially eligible, examined for eligibility, confirmed eligible, included in the study, completing follow-up, and analyzed	Pages 5-6, Methods; Figure 1
	13(b)	Give reasons for non-participation at each stage	Page 6, Methods; Figure 1
	13(c)	Consider use of a flow diagram	Page 19, Figure 1

Item	No.	Recommendation	Page/Section in Manuscript
Descriptive data	14(a)	Give characteristics of study participants (e.g., demographic, clinical, social) and information on exposures and potential confounders	Page 8-9, Results; Table 1
	14(b)	Indicate number of participants with missing data for each variable of interest	Page 6, Methods; Table 1 and 5
Outcome data	15*	Report numbers of outcome events or summary measures	Pages 9-10, Results: Table 4; Figure 2
Main results	16(a)	Give unadjusted estimates and, if applicable, confounder-adjusted estimates and their precision (e.g., 95% confidence interval). Make clear which confounders were adjusted for and why they were included	Table 2; Figure 2. No confounder-adjusted estimates were calculated
	16(b)	Report category boundaries when continuous variables were categorized	Pages 7 and 10, Methods and Results
	16(c)	If relevant, consider translating estimates of relative risk into absolute risk for a meaningful time period	N/A- no relative risk estimates were calculated
Other analyses	17	Report other analyses done—e.g., analyses of subgroups and interactions, and sensitivity analyses	Pages 9-10, Results
Key results	18	Summarise key results with reference to study objectives	Pages 11-13, Discussion
Limitations	19	Discuss limitations of the study, taking into account sources of potential bias or imprecision. Discuss both direction and magnitude of any potential bias	Pages 13-14, Discussion-Limitations
Interpretation	20	Give a cautious overall interpretation of results considering objectives, limitations, multiplicity of analyses, results from similar studies, and other relevant evidence	Pages 11-14, Discussion
Generalisability	21	Discuss the generalisability (external validity) of the study results	Pages 8-9 and 14, Results and Discussion
Funding	22	Give the source of funding and the role of the funders for the present study and, if applicable, for the original study on which the present article is based	Funding statement discloses no funding for this project or parent project

* Give information separately for exposed and unexposed groups, where applicable.

Note: Complete the final column with the manuscript page number(s), section, or other location where each item is addressed. Use “N/A” when an item is not applicable and briefly explain if helpful.
